# Glucocorticoids regulate AKR1D1 activity in human liver *in vitro* and *in vivo*

**DOI:** 10.1530/JOE-19-0473

**Published:** 2020-02-27

**Authors:** Nikolaos Nikolaou, Anastasia Arvaniti, Nathan Appanna, Anna Sharp, Beverly A Hughes, Dena Digweed, Martin J Whitaker, Richard Ross, Wiebke Arlt, Trevor M Penning, Karen Morris, Sherly George, Brian G Keevil, Leanne Hodson, Laura L Gathercole, Jeremy W Tomlinson

**Affiliations:** 1 Oxford Centre for Diabetes, Endocrinology and Metabolism, NIHR Oxford Biomedical Research Centre, University of Oxford, Churchill Hospital, Oxford, UK; 2 Department of Biological and Medical Sciences, Oxford Brookes University, Oxford, UK; 3 Institute of Metabolism and Systems Research, University of Birmingham, Edgbaston, Birmingham, UK; 4 Diurnal Ltd, Cardiff, UK; 5 Department of Oncology and Metabolism, Faculty of Medicine, Dentistry and Health, University of Sheffield, Sheffield, UK; 6 NIHR Birmingham Biomedical Research Centre, University Hospitals Birmingham NHS Foundation Trust and University of Birmingham, Birmingham, UK; 7 Department of Systems Pharmacology & Translational Therapeutics, University of Pennsylvania Perelman School of Medicine, Philadelphia, Pennsylvania, USA; 8 Biochemistry Department, Manchester University NHS Trust, Manchester, UK

**Keywords:** 5β-reductase, NAFLD, gluconeogenesis, dexamethasone, glycogen, liver

## Abstract

Steroid 5β-reductase (AKR1D1) is highly expressed in human liver where it inactivates endogenous glucocorticoids and catalyses an important step in bile acid synthesis. Endogenous and synthetic glucocorticoids are potent regulators of metabolic phenotype and play a crucial role in hepatic glucose metabolism. However, the potential of synthetic glucocorticoids to be metabolised by AKR1D1 as well as to regulate its expression and activity has not been investigated. The impact of glucocorticoids on AKR1D1 activity was assessed in human liver HepG2 and Huh7 cells; AKR1D1 expression was assessed by qPCR and Western blotting. Genetic manipulation of AKR1D1 expression was conducted in HepG2 and Huh7 cells and metabolic assessments were made using qPCR. Urinary steroid metabolite profiling in healthy volunteers was performed pre- and post-dexamethasone treatment, using gas chromatography-mass spectrometry. AKR1D1 metabolised endogenous cortisol, but cleared prednisolone and dexamethasone less efficiently. *In vitro* and *in vivo*, dexamethasone decreased AKR1D1 expression and activity, further limiting glucocorticoid clearance and augmenting action. Dexamethasone enhanced gluconeogenic and glycogen synthesis gene expression in liver cell models and these changes were mirrored by genetic knockdown of AKR1D1 expression. The effects of AKR1D1 knockdown were mediated through multiple nuclear hormone receptors, including the glucocorticoid, pregnane X and farnesoid X receptors. Glucocorticoids down-regulate AKR1D1 expression and activity and thereby reduce glucocorticoid clearance. In addition, AKR1D1 down-regulation alters the activation of multiple nuclear hormone receptors to drive changes in gluconeogenic and glycogen synthesis gene expression profiles, which may exacerbate the adverse impact of exogenous glucocorticoids.

## Introduction

Glucocorticoids (GCs) are steroid hormones that are released in response to stress and play a crucial role in inflammation and in carbohydrate, lipid and protein metabolism. Within key metabolic target tissues, notably the liver, the availability of GCs to bind and activate the GC receptor (GR) is controlled by a series of pre-receptor enzymes that inactivate or regenerate active GCs. In this regard, the role of the 11β-hydroxysteroid dehydrogenases (11β-HSD, type 1 and 2) and the 5α-reductases (type 1 and 2) are well established (Morgan *et al.* 2014, Nasiri *et al.* 2015). We have recently shown that 5β-reductase (AKR1D1) is also a potent regulator of GC availability and GR activation in human hepatocytes (Nikolaou *et al.* 2019*a*).

AKR1D1 is a member of the aldo-keto-reductase (AKR) superfamily 1 of enzymes and is the first member of the 1D subfamily (Onishi *et al.* 1991, Faucher *et al.* 2008). The human gene consists of nine exons and six transcript variants that have been identified, three of which lead to functional protein isoforms. AKR1D1 is principally expressed in the liver, where levels are more than ten-fold higher than in any other tissue (Chen & Penning 2014). In addition to governing GC availability (as well as the availability of other steroid hormones including progesterone and androgens) (Kondo *et al.* 1994, Chen *et al.* 2011, Nikolaou *et al.* 2019*a*), we have shown that AKR1D1 has an important role in regulating lipid metabolism in human hepatocytes, largely, although not exclusively, through its role to limit the generation of bile acids (BAs) that can activate the farnesoid X receptor (FXR) (Nikolaou *et al.* 2019*b*).

However, important questions remain unanswered regarding the role of AKR1D1 in GC metabolism, specificially with regard to regulation of AKR1D1 expression and activity by GCs, the capacity of AKR1D1 to metabolise synthetic steroids and its role in the regulation of established GC target genes. There is a precedent for GCs regulating their own pre-receptor metabolism. GCs are known to increase 11β-HSD1 activity and expression and this has been postulated as a mechanism driving local GC excess and fueling an adverse metabolic phenotype (Jamieson *et al.* 1995, Dube *et al.* 2015). While the differential feedback of BAs to regulate AKR1D1 expression has been previously described (Valanejad *et al.* 2017), to date, the interplay between GCs and AKR1D1 expression and activity has not been explored.

Our study therefore had two major aims; first, to examine the potential for GCs to regulate AKR1D1 expression and activity and, secondly, to determine if established GC sensitive molecular targets are also regulated by changes in AKR1D1 and, if so, whether this is mediated through GR or non-GR mediated mechanisms. 

## Materials and methods

### Cell culture

HepG2 cells (Cat#HB-8065) and HEK293 cells (Cat#CRL-11268) were purchased from ATCC. Huh7 cells were purchased from the Japanese Cancer Research Resources Bank (Cat#JCRB0403). All cell lines were cultured in Dulbecco’s minimum essential medium (DMEM) (Thermo Fisher Scientific), containing 4.5 g/L glucose and supplemented with 10% fetal bovine serum, 1% penicillin/streptomycin and 1% non-essential amino acids (Thermo Fisher Scientific).

Dexamethasone (500 nM), cortisol (500 nM), prednisolone (500 nM), GW4064 (5 μM), GSK2033 (100 nM), 22(S)-hydroxycholesterol (10 μM) and RU486 (5 μM) were purchased from Sigma-Aldrich. SPA70 (10 μM) was purchased from Axon Medchem (Groningen, Netherlands). For all cell treatments, HEK293, HepG2 and Huh7 cells were cultured in serum-free and phenol red-free media containing 4.5g/L glucose and supplemented with 10% fetal bovine serum, 1% penicillin/streptomycin and 1% non-essential amino acids.

### Transfection studies

AKR1D1 over-expression studies were performed in 12-well cell bind plates (Corning). The pCMV6-XL4 + AKR1D1 (Origene Technologies, Rockville, MD, USA) construct was used and 0.5 μg DNA and 1 μL X-tremeGENE DNA transfection reagent (Roche) were diluted in 100 μL OPTIMEM serum-free media (Invitrogen). The mixture was vortexed and incubated at room temperature for 20 min and, subsequently, 100 μL was added to each well and cells were incubated at 37°C for 48 h prior to treatment.

For AKR1D1 knockdown studies, cells were plated in 24-well cell bind plates (Corning). AKR1D1 siRNA molecules (HSS1101183, HSS1101184) were purchased from Invitrogen. 20 nmol of AKR1D1 siRNA was diluted in 25 μL OPTIMEM serum-free media (Invitrogen) and, in a separate tube, 2.5 μL Lipofectamine RNAiMAX (Invitrogen) was diluted in 25 μL OPTIMEM serum-free media. The contents of the two tubes were combined by gentle pipetting and incubated at room temperature for 5 min. 50 μL of the resulting transfection solution was added drop-wise and cells were incubated at 37°C for 48 h prior to treatment.

### Luciferase reporter assay

To determine GR activation, HEK293 cells were plated in 24-well cell bind plates (Corning) and co-transfected with AKR1D1 over-expression vector (as described above) and GRE-reporter: a mixture of an inducible GRE-responsive firefly luciferase construct and a constitutively expressing renilla luciferase construct (#CCS-006L, Qiagen). Cell lysates were harvested in passive lysis buffer, and reporter activity was measured using the Luciferase Assay System (Promega) and an EnSpire Multimode plate reader (PerkinElmer). The data were presented as the percentage ratio of firefly to renilla luciferase activity (Fluc/Rluc).

### RNA extraction and gene expression (quantitative PCR)

Total RNA was extracted from cells using the Tri-Reagent system (Sigma-Aldrich), and RNA concentrations were determined spectrophotometrically at OD260 on a Nanodrop spectrophotometer (ThermoFisher Scientific). RT was performed in a 20 μL volume; 1 μg of total RNA was incubated with 10× RT Buffer, 100 mM dNTP Mix, 10× RT Random Primers, 50 U/μL MultiScribe Reverse Transcriptase and 20 U/μL RNase Inhibitor (ThermoFisher Scientific). The reaction was performed under the following conditions; 25°C for 10 min, 37°C for 120 min and then terminated by heating to 85°C for 5 min.

All quantitative PCR (qPCR) experiments were conducted using an ABI 7900HT sequence detection system (Perkin-Elmer Applied Biosystems). Reactions were performed in 6 μL volumes on 384-well plates in reaction buffer containing 3 μL of 2× Kapa Probe Fast qPCR Master Mix (Sigma-Aldrich). All probes were supplied by Thermo Fisher Scientific as predesigned TaqMan Gene Expression Assays (FAM dye-labeled). The reaction conditions were 95°C for 3 min, then 40 cycles of 95°C for 3 s and 60°C for 20 s. The Ct (dCt) of each sample using the following calculation (where E is reaction efficiency, determined from a standard curve): ΔCt = E^(min Ct−sample Ct)^ using the 1/40 dilution from a standard curve generated from a pool of all cDNAs as the calibrator for all samples. The relative expression ratio was calculated using the following formula: Ratio = ΔCt_(target)_/ΔCt_(ref)_ and expression values were normalized to 18SrRNA (Pfaffl 2001).

### Protein extraction and immunoblotting

Total protein was extracted from cells using RIPA buffer (150 mM NaCl, 1.0% IGEPAL® CA-630, 0.5% sodium deoxycholate, 0.1% SDS, and 50 mM Tris, pH 8.0) (Sigma-Aldrich) and protease inhibitor cocktail (Thermo Fisher Scientific). Protein concentrations were measured using a commercially available assay (Bio-Rad Laboratories) according to the manufacturer’s protocol. Primary human AKR1D1 (dilution 1/250; HPA057002, Atlas Antibodies AB, Bromma, Sweden), GILZ (sc-133215, Santa Cruz Biotechnology), β-tubulin (#15115, monoclonal) (Cell Signaling), β-actin (#3700, monoclonal) (Cell Signaling), CYP8B1 (#PA5-37088, polyclonal) (ThermoFisher Scientific) and secondary antibodies (P044801-2, polyclonal) from Dako (Agilent) were used at a dilution of 1/1000 (primary) and 1/2000 (secondary) respectively, unless stated otherwise. Bands were visualised with Bio Rad Clarity Western ECL and ChemiDocXS imager (Bio Rad). Western blots were quantified by densitometry analysis using ImageJ (https://imagej.nih.gov/ij/), normalised to β-tubulin to correct for variability in gel loading.

### Clinical protocol

The study was approved by the South East Wales Research Ethics Committee, and all participants gave written informed consent. The study protocol was authorised by the Medicines and Healthcare products Regulatory Agency (EudraCT number: 2013-000259-42). Fourteen healthy male participants with no significant past medical history and who were on no regular prescribed medication were recruited into the study and investigated on two occasions. On their first assessment, participants performed a timed (8 h) urine collection starting at 24:00 h and ending at 08:00 h the following morning. On their second assessment, they took dexamethasone 1 mg at 23:00 h, and then performed the timed urine collection from 24:00 to 08:00 h as before. Urine collection aliquots were stored at −20°C until analysis by gas chromatography-mass spectrometry as described.

### Steroid hormone measurements

For *in vitro* media steroid hormone treatments, quantitative gas chromatography-mass spectrometry (GC-MS) was undertaken in selected ion-monitoring analysis mode as described previously (Shackleton 1986). An Agilent 5973 instrument was used in a selected ion monitoring mode and the following steroids were identified: cortisol, cortisone, 5β-tetrahydrocortisone (5β-THE), 5β-tetrahydrocortisol (5β-THF), 5α-tetrahydrocortisol (5α-THF) and cortisol-d4. Cortisol was positively identified by comparison to an authentic reference standard that gave a double peak at approximately 24.17 min, monitored ion was 605. Cortisone was positively identified by comparison to an authentic reference standard that gave a double peak at approximately 23.20 min, monitored ion was ion 531. The monitored ions for 5β-THE and 5β-THF were 578 and 562, respectively, and were positively identified at approximately 18.87 min and 19.95 min, respectively. In selected experiments, cell media cortisone levels were also determined using a commercially available cortisone ELISA assay (<0.1% cross-reactivity with dexamethasone), according to the manufacturer’s protocol (Invitrogen). Cell media prednisolone and dexamethasone were measured by liquid chromatography-mass spectrometry (LC-MS/MS) using previously published methods (Owen *et al.* 2005, Hawley *et al.* 2018). The lower limit of quantitation was 5.2 nmol/L and 0.25 nmol/L for prednisolone and dexamethasone, respectively.

Urinary corticosteroid metabolite analysis was performed by GC-MS, as described previously (Shackleton 1986, Palermo *et al.* 1996). Total cortisol metabolites were defined as the sum of cortisol, 6-OH-cortisol, cortisone, 5β-THF, 5α-THF, 5β-THE, α-cortolone, β-cortolone, α-cortol and β-cortol. 5β-THF is the 5β-reduced metabolite generated by AKR1D1, whilst 5α-THF is generated through the activity of 5α-reductases (type 1 and 2). The 5β-THF/5α-THF ratio provides a measure of the relative activity of AKR1D1 and 5α-reductases.

### Statistics

Data are presented as mean±s.e., unless otherwise stated. Normal distribution was confirmed using Shapiro–Wilk test. Two-tailed, paired *t*-tests were used to compare single treatments to control. For comparisons between control and different treatments, statistical analysis was performed using one-way ANOVA with Dunnett corrections. To compare mean differences between groups that had been split on multiple treatments, doses or times, two-way ANOVA with Sidak corrections was used. Statistical analysis on qPCR data was performed on mean relative expression ratio values (Ratio = ΔCt(target)/ΔCt (Pfaffl 2001)). Data analysis was performed using Graphpad Prism software (Graphpad Software Inc) and considered statistically significant at *P* < 0.05, unless otherwise stated.

## Results

### AKR1D1 differentially regulates endogenous and synthetic glucocorticoid clearance *in vitro*

We first explored the capacity of AKR1D1 to metabolise endogenous and synthetic GCs. HEK293 cells were transfected with either empty pCMV6-XL4 vector (EV) or *AKR1D1* containing vector (Origene Technologies) for 48 h. Successful over-expression was confirmed using qPCR and Western blotting (Supplementary Fig. 1A and B, see section on supplementary materials given at the end of this article).

**Figure 1:**
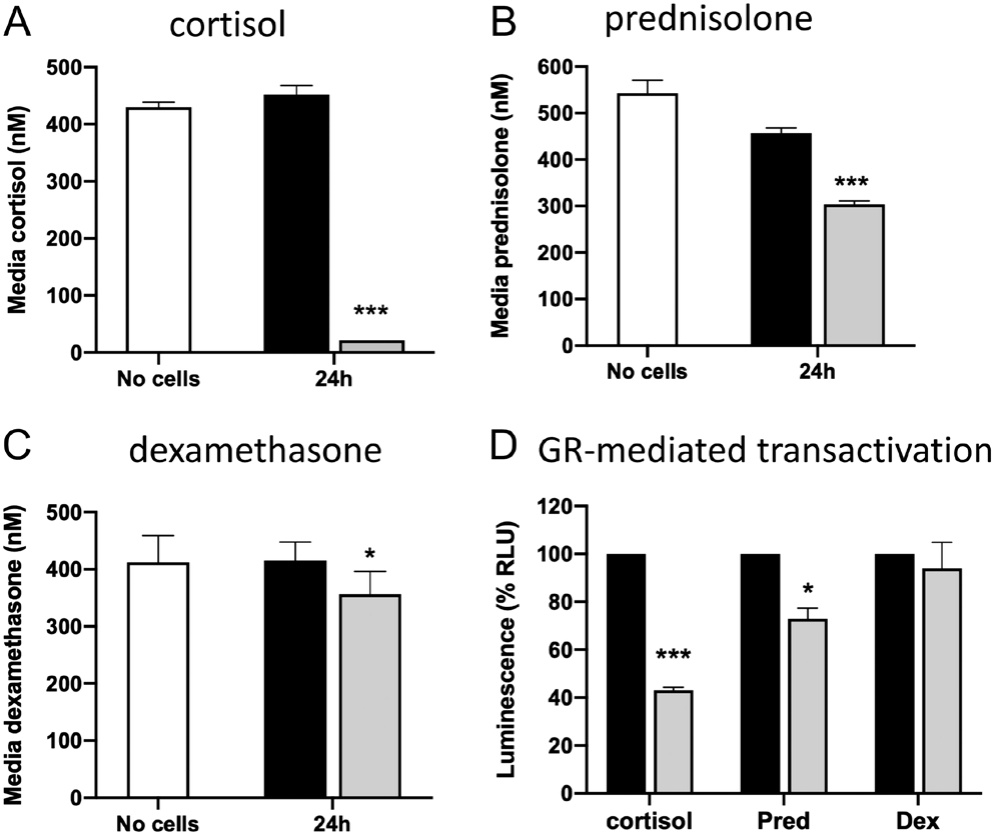
AKR1D1 differentially regulates endogenous and synthetic GC metabolism *in vitro*. AKR1D1 over-expression (grey bars) increases cortisol (A) and prednisolone clearance (B), following 24 h of treatment, compared to no-cell controls (white bars) or vector only transfected cells (black bars). AKR1D1 over-expression had a limited, although significant effect on dexamethasone clearance, following 24 h of treatment, compared to no-cell controls (white bars) or vector only transfected cells (black bars) (C). AKR1D1 over-expression (grey bars) significantly decreased activation of the glucocorticoid receptor in HEK293 cells, following cortisol and prednisolone treatment, but not following dexamethasone treatment (all 500 nM, 24 h), as measured by activation of GRE-luciferase-reporter (D). Firefly luciferase activity was normalised to renilla luciferase. Data are presented as mean±s.e. of *n* = 8 experiments, performed in duplicate. *P < 0.05, ***P < 0.001, compared to vector only transfected controls.

Following *AKR1D1* over-expression, HEK293 cells were treated with cortisol, prednisolone or dexamethasone (500 nM, 24 h) and cell media GC concentrations measured using mass-spectrometry. Cortisol was almost completely cleared within 24 h in cells over-expressing *AKR1D1* in comparison with empty vector controls (Fig. 1A). In contrast, there was only partial clearance of prednisolone (33%) and dexamethasone (15%) (Fig. 1B and C). To determine the impact of these observations on GR activation, dual transfection experiments were performed. HEK293 cells were transfected with both the *AKR1D1* expressing vector and a commercially available GR-element (GRE) luciferase construct. Consistent with the mass-spectrometry data, AKR1D1 over-expression decreased cortisol-mediated GR activation (EV: 100% vs AKR1D1: 43.1 ± 1.2%, *P* < 0.001). The impact on prednisolone-mediated GR activation was less marked, but remained significant (EV: 100% vs AKR1D1: 73.0 ± 4.4%, *P* < 0.05). There was no effect of AKR1D1 over-expression on dexamethasone-mediated GR activation (EV: 100% vs AKR1D1: 94.0 ± 10.8%, *P* = ns) (Fig. 1D).

### Cortisol fails to regulate GC target genes in human hepatoma cells due to rapid clearance

**Figure 2:**
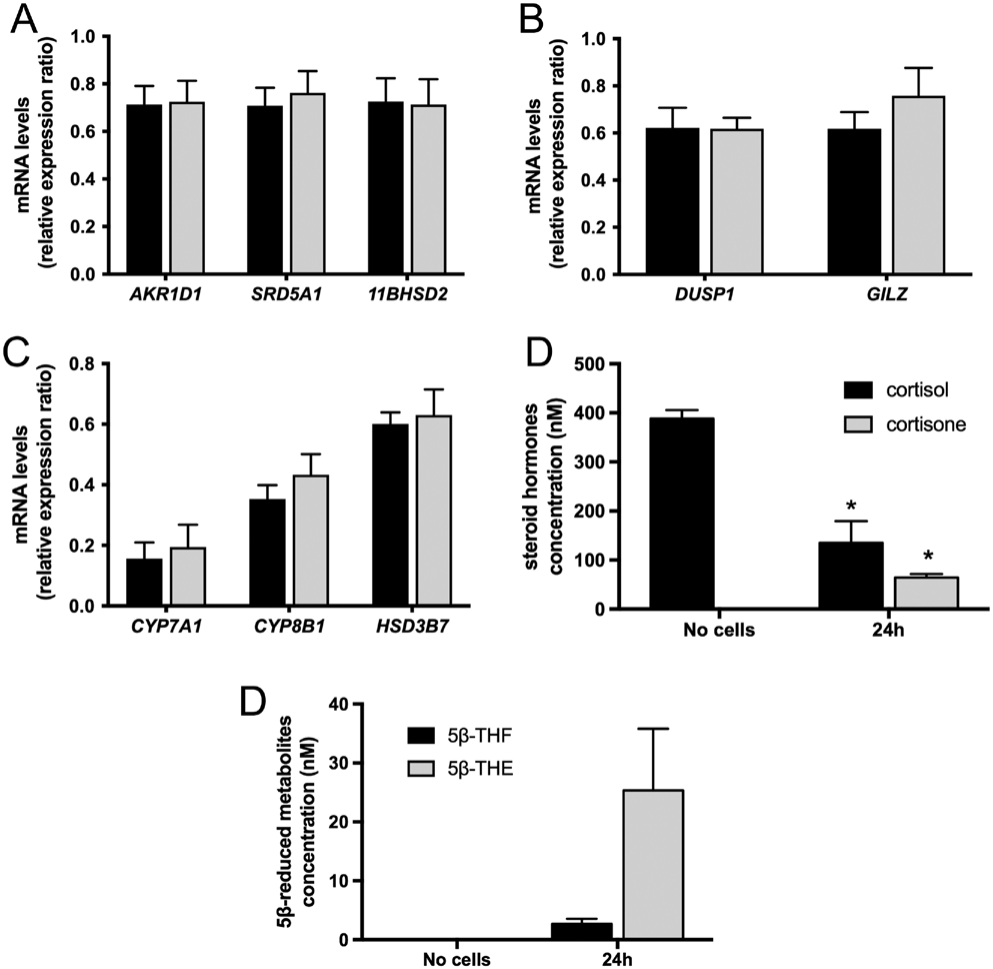
Endogenous GCs fail to regulate *AKR1D1* expression *in vitro*. Cortisol treatment of HepG2 cells (500 nM, 24 h) has no effect on the expression of steroid metabolising, glucocorticoid receptor regulated or bile acid synthesis genes (A, B and C). Mass spectrometry analysis of cell culture media demostrates increased cortisol clearance with a parallel increase in cortisone formation, indicative of 11β-HSD2 activity (D). Cell culture media 5β-tetrahydrocortisol (5β-THF) and 5β-tetrahydrocortisone (5β-THE) levels increased following cortisol treatment (500 nM, 24 h) (E). qPCR data were normalised to 18SrRNA. Data are presented as mean ± s.e. of *n* = 5 experiments, performed in triplicate, *P < 0.05, compared no-cell controls.

To further demonstrate the potent ability of human hepatoma cell lines to clear endogenous cortisol, HepG2 human hepatoma cells were treated with cortisol (500 nM, 24 h). Cortisol failed to regulate hepatic gene expression (Fig. 2A, B and C). Subsequent GC-MS analysis of the cell media demonstrated enhanced clearance of cortisol with a parallel increase in cortisone production, as a result of endogenous 5αR/5βR and 11β-HSD2 activity, respectively (Fig. 2D). As expected, the levels of 5β-reduced metabolites of cortisol and cortisone, 5β-THF and 5β-THE, increased significantly (Fig. 2E). These data suggest that increased cortisol clearance underpins the lack of effect of cortisol on gene expression in HepG2 cells.

### Dexamethasone treatment down-regulates AKR1D1 expression and activity *in vitro* and *in vivo*

**Figure 3:**
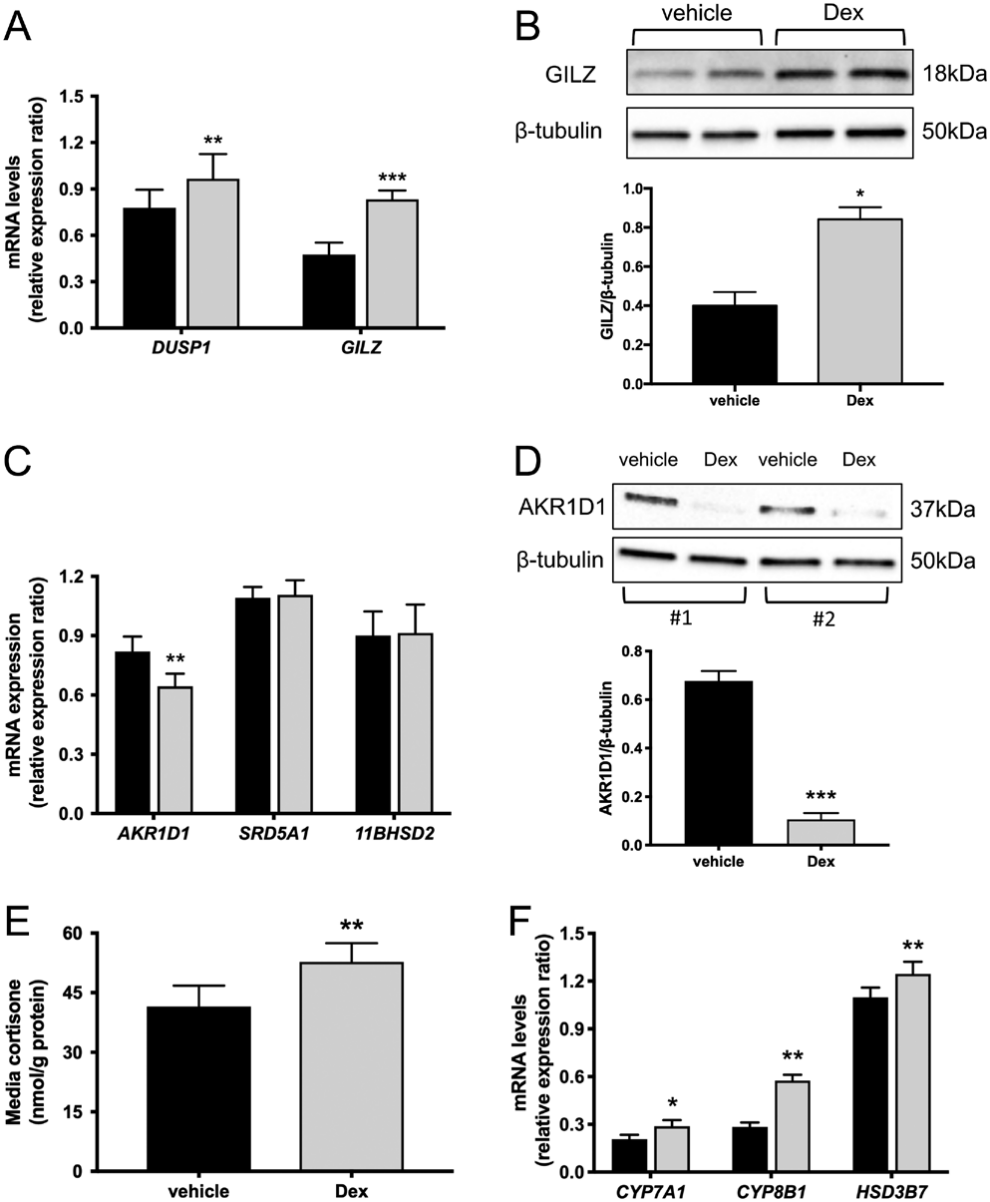
Synthetic GCs down-regulate AKR1D1 expression and activity *in vitro*. Dexamethasone treatment of HepG2 cells (500 nM, 24 h) increases the mRNA and protein expression of the glucocorticoid regulated genes, *DUSP1* and *GILZ* (A and B). Dexamethasone treatment decreases the mRNA and protein expression of AKR1D1, but it had no effect on the expression of the steroid-metabolising genes *SRD5A1* and *11BHSD2* (C and D), with a concomitant decrease in cortisone clearance, following 8 h of cortisone treatment (200 nM) (E). Dexamethasone treatment increases the expression of the bile acid synthesis genes *CYP7A1*, *CYP8B1* and *HSD3B7* (F). Representative Western blot images are shown, and formal quantification was performed in *n* = 5 replicates. qPCR data were normalised to 18SrRNA. Data are presented as mean ± s.e. of *n* = 5–7 experiments, performed in triplicate, *P < 0.05, **P < 0.01, ***P < 0.001, compared vehicle-treated controls.

Due to its limited clearance by AKR1D1, dexamethasone was used to examine the potential regulation of AKR1D1 activity and expression by GCs. HepG2 cells were treated with dexamethasone (500 nM) for 24 h; successful activation of the GR was confirmed by elevated mRNA levels of the GR-regulated genes *DUSP1* and *GILZ*, with a concomitant increase in GILZ protein expression (Fig. 3A and B). Dexamethasone decreased AKR1D1 mRNA and protein expression, without impacting on the expression of *SRD5A1* and *11BHSD2* (Fig. 3C and D). To assess functional AKR1D1 activity, cortisone (which is metabolised by AKR1D1 in hepatocytes) clearance (200 nM, 8 h) was measured in cells that had been treated with dexamethasone. Paralleling the gene expression data, dexamethasone limited cortisone clearance in HepG2 cells, consistent with decreased AKR1D1 expression (Fig. 2E). In addition to regulating AKR1D1, dexamethasone increased the expression of other key genes involved in the BA synthetic pathway, including *CYP7A1*, *CYP8B1* and *HSD3B7* (Fig. 3F).

**Figure 4:**
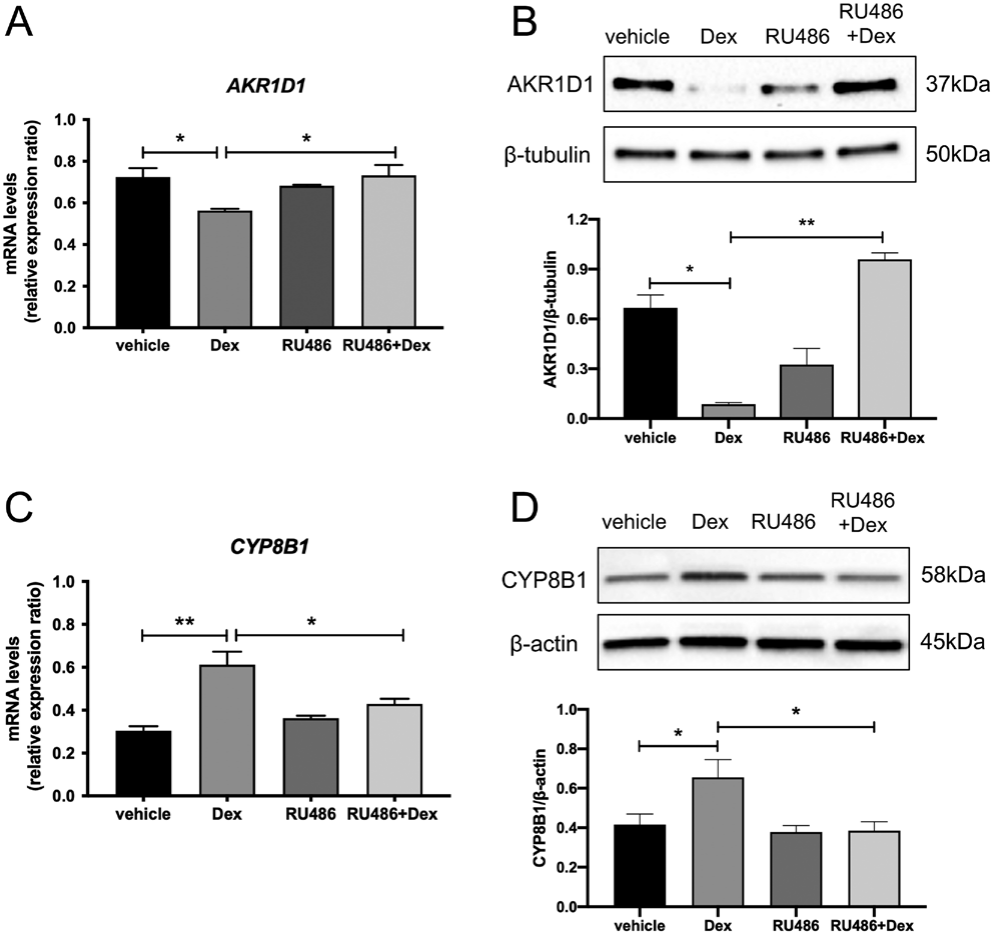
GCs regulate AKR1D1 expression through GR activation. Dexamethasone treatment decreases AKR1D1 mRNA (A) and protein expression (B). Addition of the glucocorticoid receptor antagonist RU486 (5 μM, 24 h) in the dexamethasone-treated HepG2 cells normalises the expression levels of AKR1D1 (A and B). RU486 also normalises the dexamethasone-induced expression of CYP8B1 (C and D). Representative Western blot images are shown, and formal quantification was performed in *n* = 5 replicates. Representative Western blot images are shown, and formal quantification was performed in *n* = 5 replicates. qPCR data were normalised to 18SrRNA. Data are presented as mean±s.e. of *n* = 5 experiments, performed in triplicate, *P < 0.05, **P < 0.01, ***P < 0.001, compared to vehicle-treated controls.

GILZ mRNA expression was increased following treatment with dexamethasone (500 nM, 24 h) and, as expected, this was abolished following co-treatment with RU486 (5 μM, 24 h) (Supplementary Fig. 2A). In a similar manner, the down-regulation of AKR1D1 by dexamethasone (both mRNA and protein) was reversed by co-treatment with RU486 (Fig. 4A and B), indicative of a GR-dependent mechanism. RU486 treatment also prevented the dexamethasone-induced increased expression of *CYP7A1* and *CYP8B1* (Fig. 4C, D and Supplementary Fig. 2B).

Additional experiments were performed in Huh7 human hepatoma cells. Similar patterns of gene expression changes were observed with decreased *AKR1D1* and increased *CYP7A1*, *CYP8B1* and *HSD3B7* mRNA levels following dexamethasone treatment (500 nM, 24 h). The data are summarised in Table 1.

**Table 1:** mRNA expression analysis following 24 h dexamethasone treatment in Huh7 cells.

Gene	Vehicle	Dexamethasone	*P*-value
*AKR1D1*	0.81 ± 0.04	0.66 ± 0.04^b^	<0.001
*GILZ*	1.15 ± 0.12	1.25 ± 0.11^a^	0.037
*DUSP1*	1.05 ± 0.15	1.14 ± 0.17^a^	0.024
*CYP7A1*	0.92 ± 0.04	1.08 ± 0.02^a^	0.021
*CYP8B1*	0.44 ± 0.1	0.70 ± 0.12^b^	<0.001
*HSD3B7*	0.56 ± 0.05	0.69 ± 0.06^a^	0.015
*11BHSD2*	0.52 ± 0.10	0.50 ± 0.11	0.613

Dexamethasone treatment (500 nM, 24 h) significantly decreases the expression of *AKR1D1* and increases the expression of *GILZ*, *DUSP1*, *CYP7A1*, *CYP8B1* and *HSD3B7* in Huh7 human hepatoma cells. qPCR data were normalised to 18SrRNA. Data are presented as mean±s.e. of *n* = 5 experiments, performed in triplicate, ^a^*P* < 0.05, ^b^*P* < 0.001, compared to vehicle-treated controls.

To determine if our *in vitro* observations had relevance *in vivo*, we examined urinary steroid profiles in an overnight timed (8 h) urine collection from 14 healthy male participants (age: 32.9 ± 3.1 years, BMI: 24.7 ± 0.5 kg/m^2^) investigated on two occasions, one with and one without dexamethasone treatment (1 mg), administered at the start of the timed urine collection.

**Figure 5:**
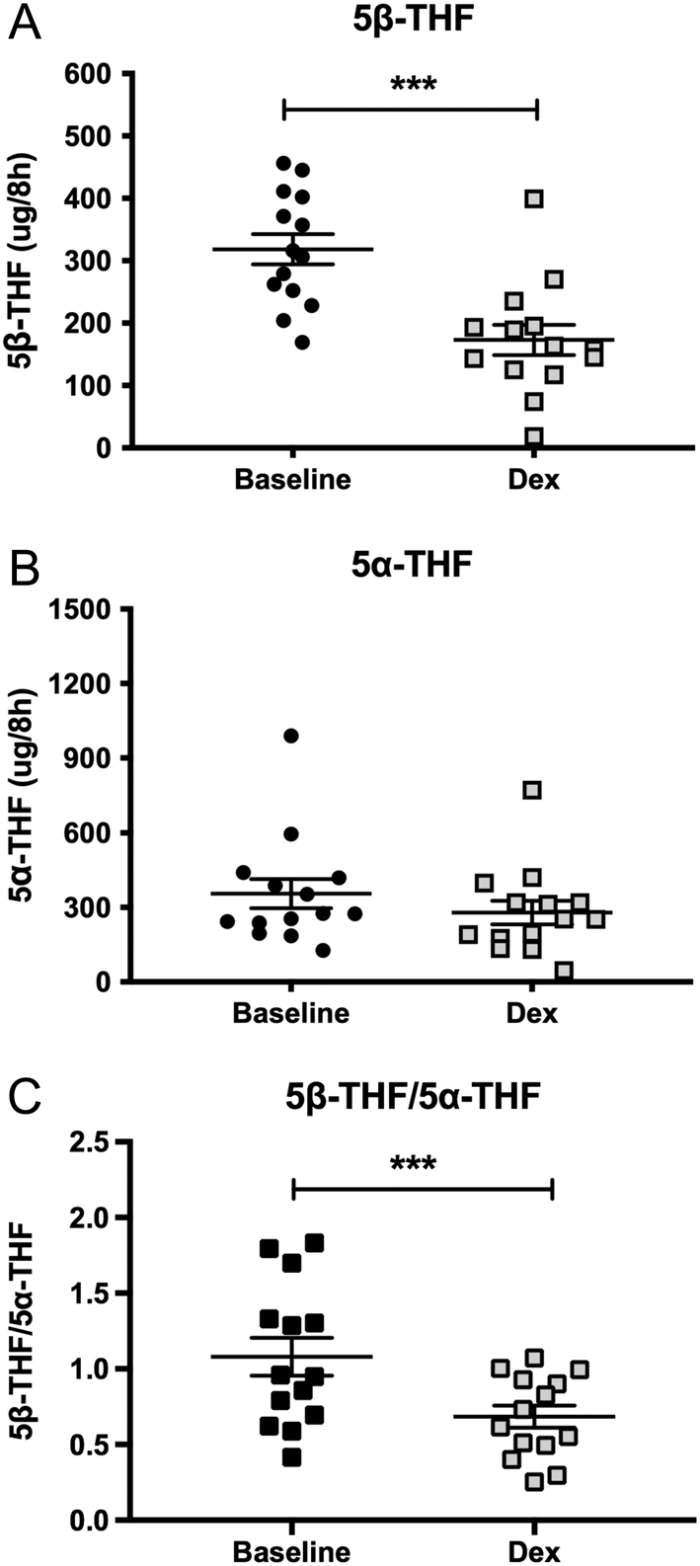
Synthetic GCs down-regulate AKR1D1 activity *in vivo*. Urine 5β-tetrahydrocortisol (5β-THF) levels decrease following over-night dexamethasone treatment, compared to overnight samples without treatment (A). There is no alteration in 5α-tetrahydrocortisol (5α-THF) levels (B). The 5β-THF/5α-THF ratio decreased following dexamethasone treatment, indicative of decreased AKR1D1 activity (C). Data are presented as mean ± s.e. of *n* = 14 participants, ***P < 0.001.

As expected, total cortisol metabolites decreased following dexamethasone treatment consistent with suppression of the hypothalamo-pituitary-adrenal axis (1898 ± 162 vs 1308 ± 135 μg/8 h, *P* < 0.01). While there was no change in 5α-THF levels, the production of the 5β-reduced metabolite of cortisol, 5β-THF, decreased following dexamethasone treatment (Fig. 5A and B). The 5β-THF/5α-THF ratio also decreased (Fig. 5C), data consistent with a dexamethasone-mediated selective reduction in AKR1D1 activity with no impact on 5α-reductase activity.

### *AKR1D1* knockdown alters glucose metabolism gene expression through FXR, GR, and PXR-dependent mechanisms

GCs have a profound effect on carbohydrate metabolism through upregulation of hepatic gluconeogenesis and glycogen synthesis (Sistare & Haynes 1985, Schneiter & Tappy 1997, Tounian *et al.* 1997). Dexamethasone treatment of HepG2 cells (500 nM, 24 h) increased mRNA expression related to these two processes, namely phosphoenolpyruvate carboxykinase (*PEPCK*), pyruvate carboxylase (*PC*), fructose-bisphosphatase 1 (*FBP1*) and glycogen synthase (*GYS1*) mRNA expression (Supplementary Fig. 2C).

**Figure 6:**
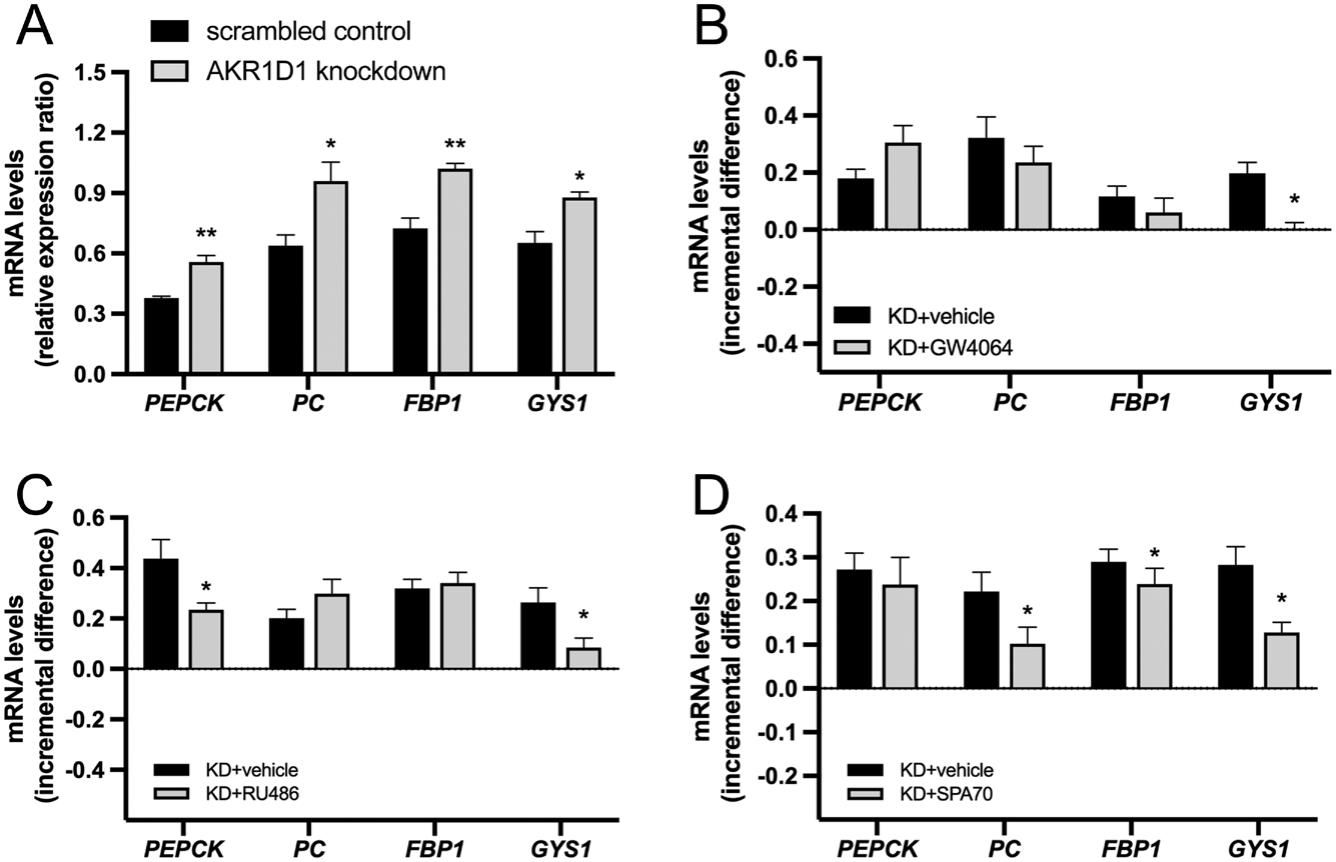
*AKR1D1* silencing drives hepatic gluconeogenic and glycogenic gene expression. *AKR1D1* knockdown (grey bars) increases the expression of *PEPCK*, *PC*, *FBP1* and *GYS1* (A). GW4064 treatment (FXR agonist: 5 μM, 24 h) normalises the expression of GYS1 in *AKR1D1* knockdown cells to levels seen in scrambled controls (B). RU486 treatment (GR antagonist: 5 μM, 24 h) limits the increase in the expression of *PEPCK and GYS1* in *AKR1D1* knockdown cells (C). The PXR antagonist, SPA70 (10 μM, 24 h), limits the increase in the expression of *PC*, *FBP1* and *GYS1* seen in *AKR1D1* knockdown cells (D). Representative Western blot images are shown, and formal quantification was performed in *n* = 5 replicates. qPCR data were normalised to 18SrRNA. Data are presented as mean ± s.e. of *n* = 5 experiments, performed in triplicate, *P < 0.05, **P < 0.01, ***P < 0.001, compared to vehicle-treated or scrambled-transfected controls. KD, AKR1D1 knockdown.

Successful *AKR1D1* knockdown in HepG2 cells was achieved using siRNA techniques (*AKR1D1* siRNA variant HSS1101183, Suppementary Fig. 3A and B). Mirroring the impact of dexamethasone treatment, and in the absence of steroid hormone supplementation in the cell media, *AKR1D1* knockdown also increased the expression of *PEPCK*, *PC*, *FBP1* and* GYS1* (Fig. 6A). To confirm that the effect of *AKR1D1* knockdown on gluconeogenic gene expression is not siRNA specific, additional experiments using a second siRNA variant (HSS1101184) were performed in HepG2 cells. The results revealed similar upregulation of *PEPCK*, *PC* and *FBP1* expression, following *AKR1D1* knockdown (Supplementary Fig. 3C and D). Additional *AKR1D1* knockdown experiments were also performed in Huh7 cells, revealing similar changes in gene expression with increased *PEPCK*, *PC* and *FBP1* mRNA levels, following *AKR1D1* knockdown. The data are summarised in Table 2.

**Table 2:** mRNA expression analysis of gluconeogenic and glycogen synthesis genes in Huh7 cells, following *AKR1D1* knockdown.

Gene	Scrambled control	*AKR1D1* knockdown	*P*-value
*AKR1D1*	0.87 ± 0.11	0.09 ± 0.01^a^	0.006
*PEPCK*	0.59 ± 0.09	0.71 ± 0.08^b^	<0.001
*PC*	0.74 ± 0.05	0.96 ± 0.05^a^	0.003
*FBP1*	0.18 ± 0.006	0.32 ± 0.01^a^	0.005
*GYS1*	0.78 ± 0.11	0.87 ± 0.12	0.22

*AKR1D1* knockdown significantly increases the expression of *PEPCK*, *PC* and* FBP1* in Huh7 human hepatoma cells. qPCR data were normalised to 18SrRNA. Data are presented as mean ± s.e. of *n* = 4 experiments, performed in duplicate, ^a^*P* < 0.01, ^b^*P* < 0.001, compared to scrambled-transfected controls.

*AKR1D1* knockdown has been previously shown to result in alterations in both FXR and LXR activation, due to decreases in primary BA synthesis and increases in oxysterol accumulation, respectively (Janowski *et al.* 1996, Nikolaou *et al.* 2019*b*). We proposed that FXR agonism and/or LXR antagonism would have the potential to rescue the phenotype in our cells. Cell treatments using the FXR agonist GW4064 (5μM, 24 h) normalised the expression of *GYS1* to levels seen in scrambled-transfected cells, but failed to rescue the upregulation of *PEPCK*, *PC* or *FBP1* expression, caused by *AKR1D1* knockdown (Fig. 6B). Additional treatments with the LXRα and LXRβ antagonists 22(S)-Hydroxycholesterol (10 μM, 24 h) and GSK2033 (100 nM, 24 h) also failed to restore *PEPCK*, *PC or FBP1* expression, suggesting that the observed phenotype is not driven by increased LXR activation (Supplementary Fig. 4A and B).

Οxysterols and cholesterol metabolites have been recently shown to activate the GR (Voisin *et al.* 2017, Silvente-Poirot *et al.* 2018). In *AKR1D1* knockdown cells, treatments with RU486 treatment (5 μM, 24 h) limited the induction of *PEPCK* and *GYS1* levels, suggesting that this observation was mediated, at least in part, through activation of the GR. However, RU486 treatment failed to rescue the up-regulation of *PC* or *FBP1* seen in *AKR1D1* knockdown cells (Fig. 6C).

In addition to LXR and GR, oxysterols are endogenous ligands of the Pregnane-X-Receptor (PXR) (Shenoy et al. 2004*a*,*b*, Li *et al.* 2007). Treatment of *AKR1D1* knockdown cells with the PXR antagonist SPA70 (10 μM, 24 h) limited the increase in gene expression of *PC*, *FBP1* and *GYS1*, indicative of an additional PXR activation mechanism of action (Fig. 6D).

## Discussion

We show that although AKR1D1 represents a crucial step in endogenous cortisol clearance, it clears synthetic steroids poorly in comparison. We demonstrate that dexamethasone decreases expression and activity of AKR1D1 *in vitro* and *in vivo* (without any effect on 3α-HSD activity, as evidenced by the lack of change in 5α-THF levels) and, finally, we reveal that the actions of AKR1D1 to regulate the expression of genes involved in glucose metabolism are mediated through FXR, GR and PXR activation.

Synthetic GCs, including dexamethasone, prednisone and prednisolone, are frequently prescribed for a variety of oncological and inflammatory conditions (van Staa *et al.* 2000, Wooldridge *et al.* 2001, Amin *et al.* 2014). Although less efficiently cleared than cortisol, we did observe some prednisolone clearance by AKR1D1, with even more limited metabolism of dexamethasone. Considering the crucial role of AKR1D1 to metabolise endogenous cortisol and cortisone, the impaired clearance of synthetic GCs that we have observed suggests an additional mechanism (over and above potency of GR activation), through which synthetic GCs may have more potent actions (both therapeutically desirable anti-inflammatory and anti-proliferative, but also undesirable metabolic and musculoskeletal side effects).

The potential role of steroid hormones, including GCs and androgens, to regulate the expression of the A-ring reductases is poorly described and has been predominantly focused on the role of androgens, only (Berman *et al.* 1995, Torres & Ortega 2003, Li *et al.* 2011). In our study, we have demonstrated that GCs decrease hepatic AKR1D1 expression both *in vitro* and *in vivo* and that this effect is mediated by activation of the GR. It is likely that these effects are mediated through glucocorticoid response elements within the promoter of *AKR1D1*; indeed, a study from Nakamoto et al. (Nakamoto *et al.* 2017) has recently shown putative GR binding sites in the *AKR1D1* gene promoter in HepG2 cells.

Published studies have shown that over-expression of AKR1D1 regulates a variety of cytochrome P450 enzymes, including increased expression of CYP3A4 (Chaudhry *et al.* 2013). Modulation of CYP3A4 activity has a profound influence of the availability of synthetic GCs; CYP3A4 inhibition along with concomitant synthetic GC administration frequently leads to the development of iatrogenic Cushing’s syndrome (Mahlab-Guri *et al.* 2011, Bernecker *et al.* 2012). Therefore, down-regulation of AKR1D1 by GCs might lead to decreased CYP3A4 and further exacerbate the adverse effects of prescribed steroids through both CYP3A4 and AKR1D1 dependent mechanisms.

AKR1D1 is down-regulated in patients with type 2 diabetes and we have recently shown a similar decrease in expression with advancing severity of non-alcoholic fatty liver disease (NAFLD) (Valanejad *et al.* 2018, Nikolaou *et al.* 2019*b*). In this context, *AKR1D1* knockdown increased the expression of key enzymes involved in lipogenesis as well as increasing functional *de novo* lipogenesis, as measured by deuterated water incorporation into fatty acids (Nikolaou *et al.* 2019*b*). The data from our study now provide additional evidence of the adverse impact of AKR1D1 down-regulation, here to drive gluconeogenesis, with the potential to fuel hepatic glucose output. The down-regulation of AKR1D1 by synthetic steroids may therefore be an important contributing factor to the adverse metabolic features associated with their use.

Oxysterols, the oxidised derivatives of cholesterol, are predominantly, although not exclusively, produced in the liver through activity of the cytochrome P450 (CYP) enzyme family (Guillemot-Legris *et al.* 2016), and they serve as potent ligands for many nuclear receptors including the LXRs, GR, PXR and the retinoic acid receptor-related orphan receptors (RORs) (Ma & Nelson 2019). In this regard, there is compelling evidence on the role of oxysterols as important mediators of metabolic syndrome (Tremblay-Franco *et al.* 2015, Guillemot-Legris *et al.* 2016, Mutemberezi *et al.* 2016). Indeed, some oxysterols are now used as biomarkers for monitoring a variety of pathologies, including atherosclerosis, BA diarrhea and Alzheimer’s disease (Eusufzai *et al.* 1993, Wang *et al.* 2016, Zmysłowski & Szterk 2019). In our study, we were not able to directly measure cell media oxysterol levels; however, we have previously shown that *AKR1D1* knockdown results in decreased primary BA formation (Nikolaou *et al.* 2019*b*) potentially leading to increased accumulation of 7α-hydroxycholestenone and 7α,12α-dihydroxycholestenone levels (oxysterols that are AKR1D1 substrates) in the cell media.

AKR1D1 has a key role in BA synthesis and drives the formation of cholic acid and chenodeoxycholic acid. Endorcing our observations, dexamethasone has been shown to increase the expression of *CYP7A1* and *CYP8B1* in both human and rat hepatocytes (Princen *et al.* 1989, Ellis *et al.* 1998, Mörk *et al.* 2016). In rodent models, data have been conflicting; in rats and mice, treatment with dexamethasone and prednisolone, respectively, resulted in decreased BA synthesis, as measured by decreased *Cyp7a1* and *Cyp8b1* expression and decreased faecal BA excretion. However, there was enhanced enterohepatic cycling of BAs with elevated plasma BA levels and biliary BA secretion (Out *et al.* 2014, Xiao *et al.* 2016). In contrast, another study has demonstrated that dexamethasone exposure to neonatal rats increased the expression of genes involved in the synthesis and enterohepatic cycling of BAs, including *Cyp7a1*, *Cyp8b1* and sodium taurocholate co-transporting polypeptide (*Ntcp*) (Liu *et al.* 2008).

The role of GCs on hepatic gluconeogenesis and glycogen synthesis has been extensively investigated. GCs increase the transcription of the gluconeogenic genes *PEPCK*, *PC*, *FBP1* and* GYS1* and their action is predominantly conveyed through activation of the GR (Stalmans & Laloux 1979, Kuo *et al.* 2015). In our study, *AKR1D1* knockdown mimicked the cellular phenotype of GC (dexamethasone) treatment. Although we have previously demonstrated the ability of *AKR1D1* knockdown to increase hepatic intracellular glycogen storage (Nikolaou *et al.* 2019*b*), this is our first effort to elucidate the mechanistic insight of the observed phenotype. Plausible hypotheses have been that this arises as a result of either impaired FXR activation, due to reduced primary BA synthesis, or increased accumulation of oxysterols, which are able to bind to and activate the GR (Voisin *et al.* 2017, Silvente-Poirot *et al.* 2018). In *AKR1D1* knockdown cells, FXR agonism normalised *GYS1* expression only; however, we were able to partially restore the gene expression profiles through the use of the GR antagonist RU486, suggesting that some of the observed changes are also driven by GR activation. Nevertheless, RU486 treatment did not correct all the changes that were observed.

Recent studies have implicated PXR in the regulation of glucose homeostasis. *In vitro*, data have been conflicting; in Huh7 cells, PXR activation using the PXR agonist rifampicin has been shown to repress gluconeogenic gene transcription (Kodama *et al.* 2007) while, in another study using HepG2 cells, rifampicin induced *PEPCK* expression (Gotoh & Negishi 2014). The latter findings are in agreement with clinical studies, where rifampicin increases blood glucose levels in humans (Rysä *et al.* 2013, Hakkola *et al.* 2016). Consistent with this, our data revealed that the gene expression phenotype associated with *AKR1D1* knockdown was partially attributable to PXR activation.

In conclusion, we have shown that AKR1D1 poorly metabolises synthetic GCs and that synthetic GCs decrease AKR1D1 expression and activity in the liver, potentially fueling the adverse metabolic phenotype associated with their use. *In vitro*, AKR1D1 down-regulation mimics the action of GCs in driving hepatic gluconeogenesis and glycogen storage. As such, this represents an additional novel mechanism by which glucocorticoids indirectly regulate glucose metabolism highlighting, in total, the complex role of AKR1D1 to govern the activation of multiple nuclear hormone receptors, with significant implications for the regulation of metabolic phenotype within the liver.

## Supplementary Material

Figure 1 AKR1D1 expression, following over-expression HEK293 cells, as measured by qPCR (a) and western blotting (b). qPCR data were normalised to 18SrRNA. Data are presented as mean±se of n=5 experiments, performed in duplicate. Representative western blot images are shown, and formal quantification was performed in n=5 replicates. *p<0.05, compared to vector only transfected controls (EV=empty vector, O/E=over-expression)

Figure 2 Dexamethasone treatment (500nM, 24h) (grey bars) increased GILZ (a) and CYP7A1 (b) mRNA expression. Addition of the glucocorticoid receptor antagonist RU486 in the dexamethasone-treated HepG2 cells normalised the expression levels of both GILZ and CYP7A1 levels in those seen in the presence of vehicle, confirming successful manipulation of glucocorticoid receptor activation (a-b). Dexamethasone treatment induced the expression of the gluconeogenic genes PEPCK, PC, FBP1, as well as the expression of glycogen synthase (GYS1), compared to vehicle-treated HepG2 cells (black bars) (c). qPCR data were normalised to 18SrRNA. Data are presented as mean±se of n=5 experiments, performed in triplicate. *p<0.05, **p<0.01, ***p<0.001, compared to vehicle-treated controls.

Figure 3 AKR1D1 knockdown (grey bars) decreases mRNA and protein expression in HepG2 cells, as measured by qPCR and western blotting (a-b). AKR1D1 siRNA variants HSS1101183 and HSS1101184 similarly decreased AKR1D1 mRNA expression, and significantly increased the mRNA expression of PCK1, PC and FBP1 in HepG2 cells. qPCR data were normalised to 18SrRNA. Data are presented as mean±se of n=4-5 experiments, performed in triplicate, *p<0.05, **p<0.01, ***p<0.001, compared to vehicle-treated or scrambled-transfected controls.

Figure 4 Pharmacological manipulation of the oxysterol receptors LXRα and LXRβ, using the LXRα antagonist 22(S)-Hydroxycholesterol (22-S HC - 10μM, 24h) or the LXRβ antagonist GSK2033 (100nM, 24h) had no impact on the alteration of expression of PEPCK, PC, FBP1, or GYS1, caused by AKR1D1 knockdown in HepG2 cells (grey bars) (a-b). qPCR data were normalised to 18SrRNA. Data are presented as mean±se of incremental gene expression of n=5 experiments, performed in triplicate, *p<0.05, **p<0.01, ***p<0.001, compared to vehicle-treated or scrambled-transfected levels (black bars). (KD=AKR1D1 knockdown)

## Declaration of interest

The authors have nothing to declare. T M P is a consultant for Research Institute for Fragrance Materials, is a recipient of a sponsored research agreement from Forendo, and is founding director of Penzymes LLC. M J W and R R are employees and stock holders in Diurnal Ltd. D D is a consultant to Diurnal Ltd.

## Funding

This work was supported by the Medical Research Council (program grant to J W T, ref. MR/P011462/1); NIHR Oxford Biomedical Research Centre (principal investigator award to J W T); British Heart Foundation (senior fellowship to L H, ref. FS/15/56/31645); National Institute of Environmental Health Sciences (P30-ES013508 awarded to T M P); NIHR Birmingham Biomedical Research Centre (BRC-1215-20009 to W A) and the Wellcome Trust (Investigator Award 209492/Z/17/Z to W A).

## Author contribution statement

The study was developed by N N, L L G and J W T. N N, B G K, B A H, K M, S G and W A designed the methods. N N, A A, N A and A S performed the investigation. N N wrote the manuscript and it was reviewed and edited by N N, D D, M J W, R R, T M P, B G K, L H, W A and J W T. J W T performed supervision and acquired the funding.
