# Supplementary figures and images for: Glucocorticoids regulate AKR1D1 activity in human liver in vitro and in vivo
Source: J Endocrinol. 2020 Feb 27;245(2):207–18. doi: 10.1530/JOE-19-0473 (PMC7182088; doi:10.1530/JOE-19-0473)

Figure 1

a

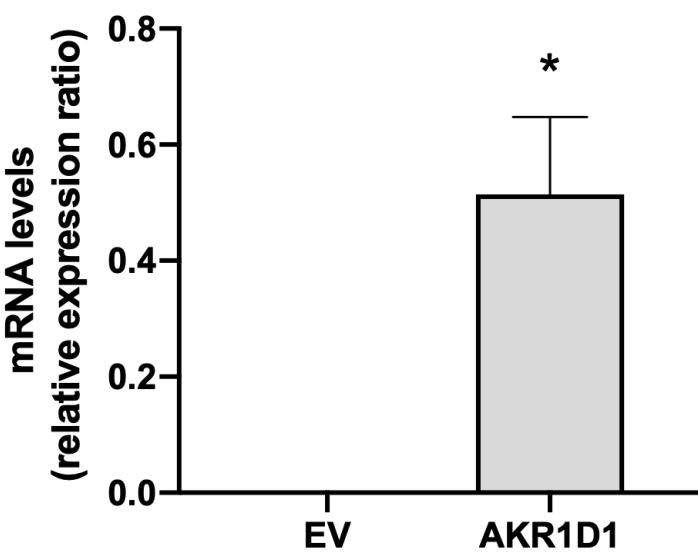

b

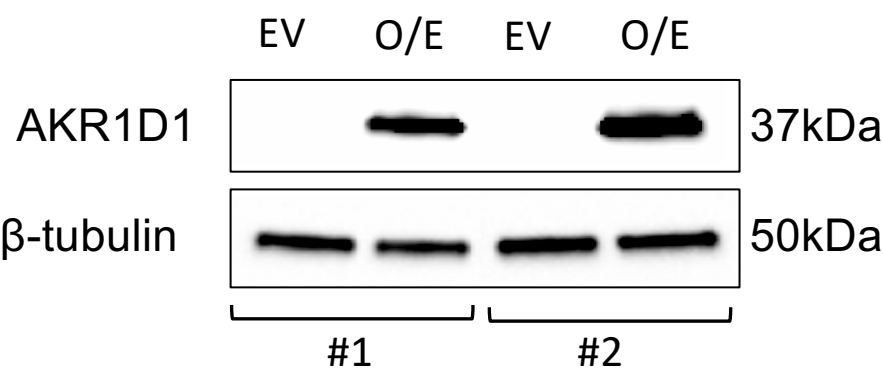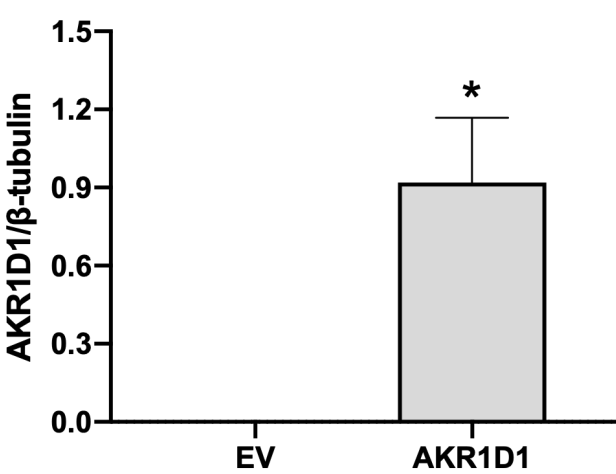

Supplement: Figure 1 AKR1D1 expression, following over-expression HEK293 cells, as measured by qPCR (a) and western blotting (b). qPCR data were normalised to 18SrRNA. Data are presented as mean±se of n=5 experiments, performed in duplicate. Representative western blot images are shown, and formal quantificatio [file supplementary_figure_1.pdf]

Figure 2

a

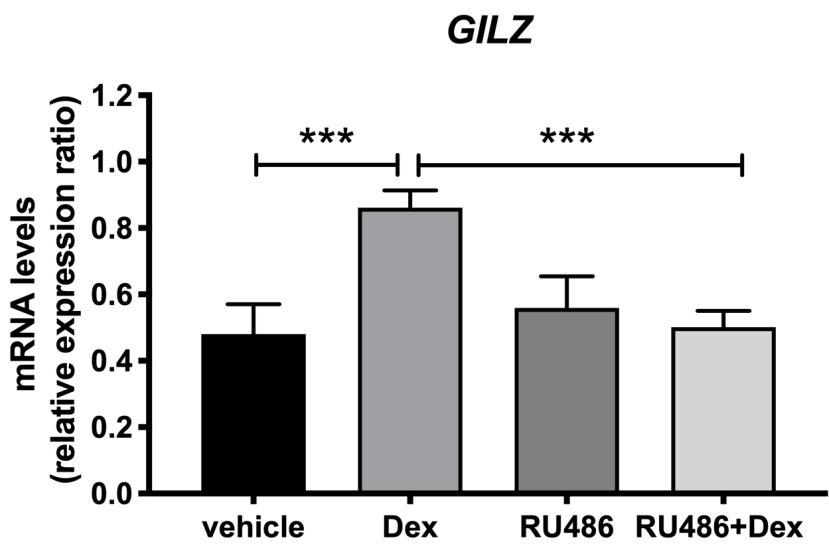

b

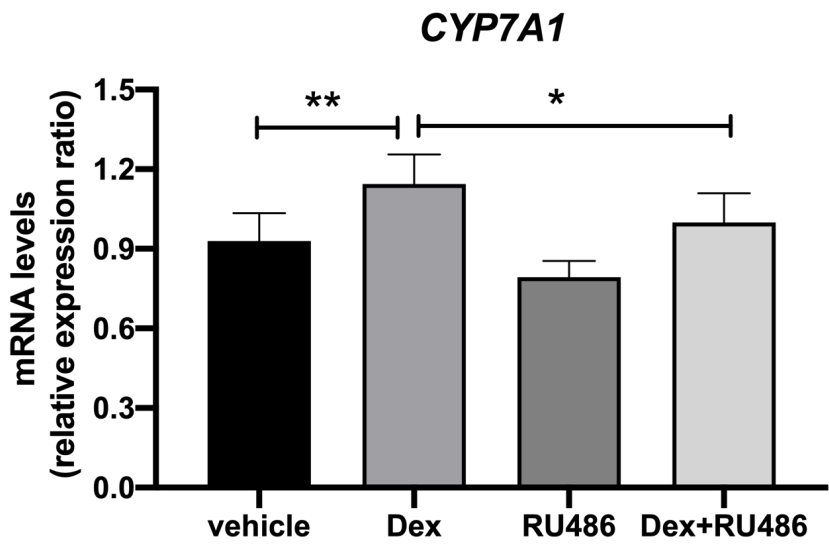

c

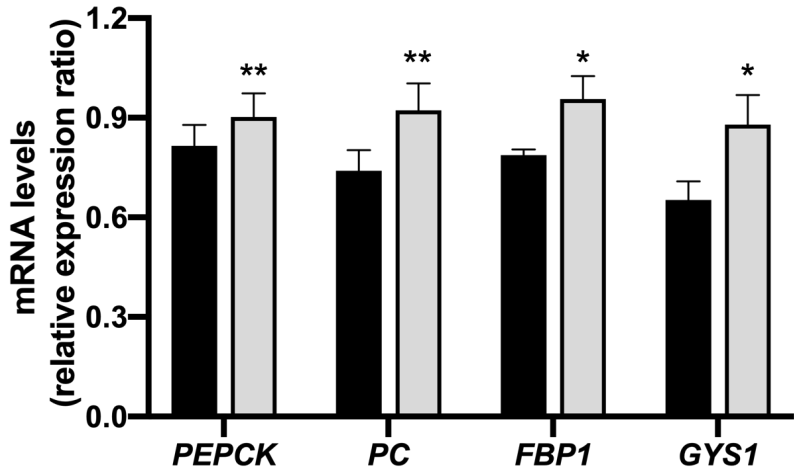

Supplement: Figure 2 Dexamethasone treatment (500nM, 24h) (grey bars) increased GILZ (a) and CYP7A1 (b) mRNA expression. Addition of the glucocorticoid receptor antagonist RU486 in the dexamethasone-treated HepG2 cells normalised the expression levels of both GILZ and CYP7A1 levels in those seen in the presence [file supplementary_figure_2.pdf]

Figure 3

a

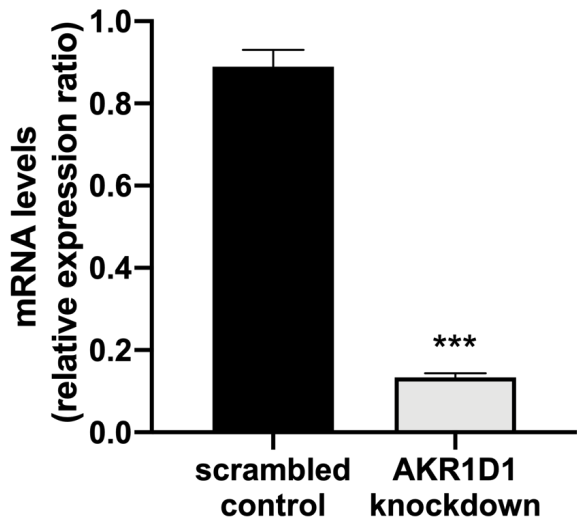

b

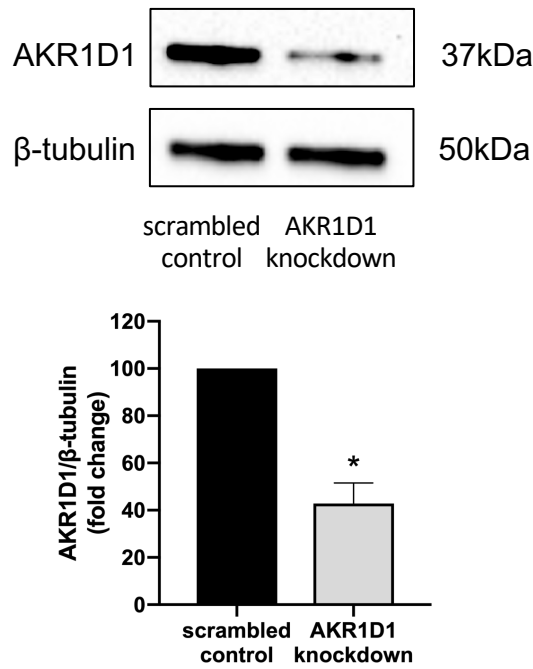

c

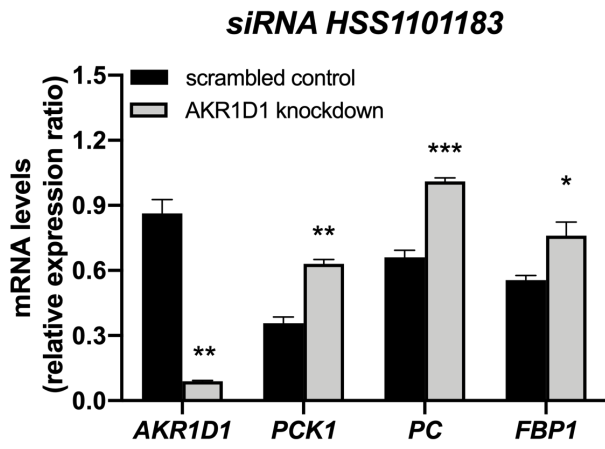

d

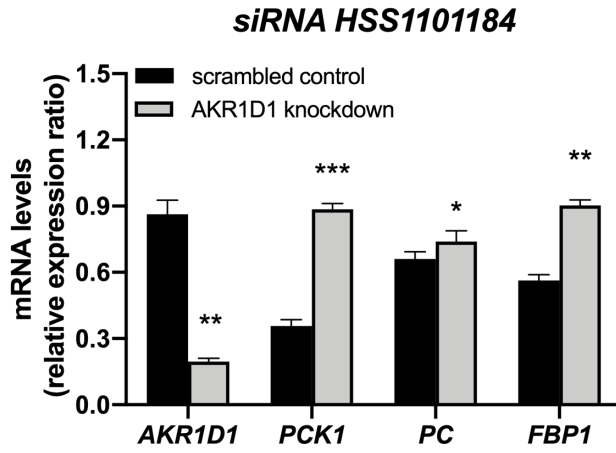

Supplement: Figure 3 AKR1D1 knockdown (grey bars) decreases mRNA and protein expression in HepG2 cells, as measured by qPCR and western blotting (a-b). AKR1D1 siRNA variants HSS1101183 and HSS1101184 similarly decreased AKR1D1 mRNA expression, and significantly increased the mRNA expression of PCK1, PC and FBP1 [file supplementary_figure_3.pdf]

Figure 4

a

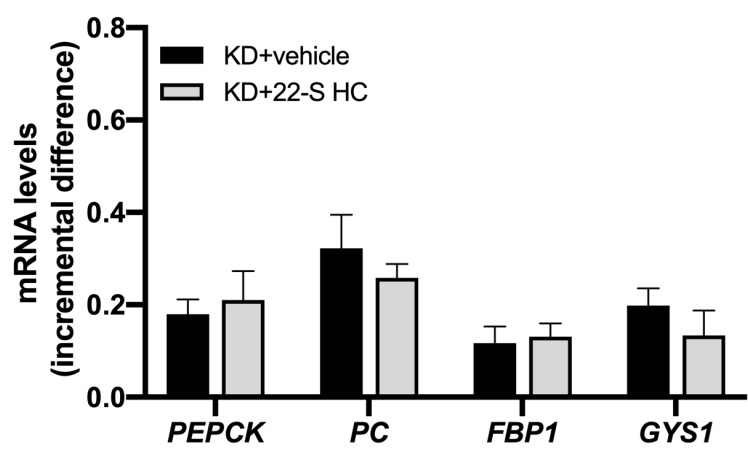

b

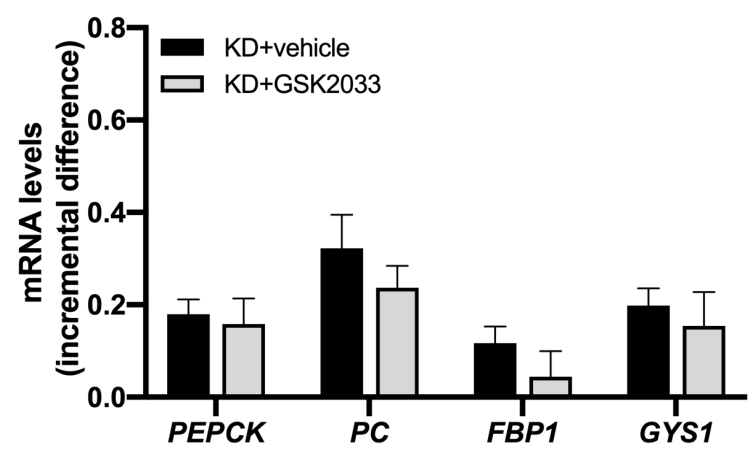

Supplement: Figure 4 Pharmacological manipulation of the oxysterol receptors LXRα and LXRβ, using the LXRα antagonist 22(S)-Hydroxycholesterol (22-S HC - 10μM, 24h) or the LXRβ antagonist GSK2033 (100nM, 24h) had no impact on the alteration of expression of PEPCK, PC, FBP1, or GYS1, caused by AKR1D1 knockdown i [file supplementary_figure_4.pdf]
